# Community temporal variability increases with fluctuating resource availability

**DOI:** 10.1038/srep45280

**Published:** 2017-03-27

**Authors:** Wei Li, M. Henry H. Stevens

**Affiliations:** 1 Yunnan Academy of Biodiversity, Southwest Forestry University, Kunming, Yunnan 650224, China; 2 Department of Biology, Miami University, Oxford, Ohio 45056, USA

## Abstract

An increase in the quantity of available resources is known to affect temporal variability of aggregate community properties. However, it is unclear how might fluctuations in resource availability alter community-level temporal variability. Here we conduct a microcosm experiment with laboratory protist community subjected to manipulated resource pulses that vary in intensity, duration and time of supply, and examine the impact of fluctuating resource availability on temporal variability of the recipient community. The results showed that the temporal variation of total protist abundance increased with the magnitude of resource pulses, as protist community receiving infrequent resource pulses (i.e., high-magnitude nutrients per pulse) was relatively more unstable than community receiving multiple resource pulses (i.e., low-magnitude nutrients per pulse), although the same total amounts of nutrients were added to each community. Meanwhile, the timing effect of fluctuating resources did not significantly alter community temporal variability. Further analysis showed that fluctuating resource availability increased community temporal variability by increasing the degree of community-wide species synchrony and decreasing the stabilizing effects of dominant species. Hence, the importance of fluctuating resource availability in influencing community stability and the regulatory mechanisms merit more attention, especially when global ecosystems are experiencing high rates of anthropogenic nutrient inputs.

Ecological stability represents a major structuring theme in ecology, and decades of efforts and endeavors by ecologists have continuously stimulated productive research. Given that ecological stability is a multidimensional concept, including spatial and temporal stability, resistance, resilience, persistence and some other components^1^,^2^,^3^, confusion may arise from these different definitions of ecological stability. Here we focus on one important type of ecological stability, the temporal stability of total community abundance. It has been widely recognized that resource availability plays a central role in regulating community temporal stability (or temporal variability, the inverse of temporal stability)^3^,^4^,^5^,^6^,^7^. For instance, theoretical models propose that resource enrichment could weaken community temporal stability by causing fluctuations in population dynamics^4^,^5^, which is corroborated by some experimental evidence^8^,^9^,^10^,^11^,^12^. Beside this direct impact, resource enrichment could also indirectly affect community stability mediated by its direct impact on species diversity^13^,^14^. Some other studies found that asynchronous responses of species to various types of environmental change play vital roles in sustaining community stability, which, however, are often independent of diversity effects on temporal stability^15^,^16^,^17^. This is because a decrease in one species’ population size might be compensated by an increase in the population size of another. As a result, such response diversity could help buffer the effects of environmental change, and community-wide asynchrony should help enhance the stability of the overall community. Additionally, component species with less variable population dynamics could contribute to less variable communities. This is particularly true when the stable presence of a few dominant species directly facilitates the process of stability propagation from the population level towards higher organizational levels^12^,^18^,^19^,^20^,^21^,^22^.

Fluctuating resources arising from climatic or environmental events are widespread phenomena in nature that have influenced fundamental ecological processes^23^,^24^. A multitude of observational and laboratory studies have shown that fluctuating resources open windows of opportunity for invaders (the fluctuating resource hypothesis)^25^,^26^,^27^,^28^,^29^,^30^, and one general accepted explanation is that fluctuating resources could minimize priority benefits shared by early-arriving resident species, and thus provide invaders windows of opportunity that would not be possible otherwise. Also, the consequences of fluctuating resource events are likely to be highly context-dependent with respect to the presence of opportunistic generalist species that can cope with environmental change, species-specific plastic responses, the type of fluctuating resources and environmental perturbations^31^,^32^,^33^,^34^. Although fluctuations in resource availability could affect, or even determine invasion success, the question of how would resident communities respond to resource pulse events remains unsolved. Specifically, given that resource pulse events occur across a wide range of ecosystems, and such events differ in pulse magnitude and duration^23^,^34^,^35^, it is surprising that a majority of studies testing the relationship between resource availability and community stability often neglect the aspect of fluctuating resource intensity, frequency and duration that characterizes such events (but see refs 36, 37, 38).

The timing of resource pulse supply relative to that of invasion occurrence might also affect resident communities. Provided that invaders are with a moderate rate of dispersal and generally exhibit similar competitive abilities as resident species, if the timing of invader arrivals coincides with that of available resource pulses so that invaders might effectively siphon off a large proportion of fluctuating resources, then their likelihood of invasion success is likely to be significantly enhanced. Meanwhile, a rapid increase in invaders’ population size might impose negative influence on resident communities. In contrast, if invaders arrive too early or too late, then resident species might seize such windows of opportunity, and put invaders in a disadvantageous position. Clearly, the influence of fluctuating resource availability on temporal variability of resident communities could be timing-dependent. However, relevant studies are limited, probably due to difficulties in controlling the magnitude and duration of resource pulse events, and in manipulating the timing of resource pulse events relative to that of invasion events in natural systems (but see refs 36 and 39, 40, 41).

Here we conducted an experiment over multiple generations in laboratory protist microcosms with multiple trophic levels. Considering that the two aspects of resource fluctuations, resource intensity and fluctuations in resource supply (i.e., resource pulse magnitude), could operate simultaneously, we added the same total amount of nutrients to each experimental microcosm to ensure that the total resource availability was constant across all microcosms. To simulate resource pulses events, we directly manipulated resource pulse magnitude so that microcosms receiving infrequent resource pulses (i.e., with a relatively short duration of resource pulse events) actually experienced larger magnitude of nutrients per pulse than microcosms receiving more frequent resource pulses (i.e., with a relatively long duration of resource pulse events). The following questions were specifically addressed: (1) Whether community temporal variability increases with enhanced resource intensity? (2) Whether community temporal variability increases with enhanced resource pulse magnitude? (3) When the timing of fluctuating resources coincides with that of invader arrivals, whether resident community displays a higher level of temporal variability than the scenario when the timing of such events is asynchronous? (4) What are the destabilizing mechanisms of resource pulses if they are causing increased temporal variability of protist community?

## Methods

The resident protist community was assembled with (5) green algal species (*Chlorella sp*., *Scenedesmus opoliensis, Cosmarium sportella, Micrasterias sp*., and *Volvox aureus*), and 5 heterotrophic protozoan species (*Colpidium Striatum, Paramecium aurelia, Euplotes eurystomus, Blepharisma americanum*, and *Spirostomum spp* (for more details about the preparation of the culture medium and the assembly of experimental microcosms see ref. 30).

We established five experimental treatments, with eight replicates for each treatment. Specifically, infrequent resource pulses (high-magnitude nutrients per pulse; treatment “Early”, “Coincident” and “Late”) were created by adding a highly concentrated medium (10.5 g protozoan pellet/L, 1.155 mL) to assigned microcosms on two consecutive days, which amounted to a nearly 130% increase in resource concentrations when compared to the control group. In contrast, frequent resource pulses (low-magnitude nutrients per pulse) were created by adding the same total amount of the concentrated medium to assigned microcosms on a daily basis over the entire experimental period (i.e., 0.055 ml of the concentrated medium per day on 42 consecutive days; treatment “Multi-pulses”). We also established a control group without adding the concentrated medium (treatment “Control”). Thus, the experimental design allowed us to directly test the effect of enhanced resource intensity on community temporal variability by comparing microcosms receiving the concentrated medium (treatment “Early”, “Coincident”, “Late” and “Multi-pulses”) with the control group (treatment “Control”), and to test the effect of enhanced resource pulse magnitude on community temporal variability by comparing microcosms experiencing high-magnitude nutrients (treatment “Early”, “Coincident” and “Late”) with microcosms experiencing low-magnitude nutrients (treatment “Multi-pulses”).

Two algal species, *Chlamydomonas reinhardtii* and *Closterium libellula*, and two protozoan species, *Tetrahymena pyriformis* and *Paramecium bursaria*, were introduced as model invaders to challenge resident protist community two weeks later after its establishment. They were similar in body size to resident species, and none of them fed at multiple trophic levels, or was found in the pre-invasion community. Our previous study examined the effects of resource pulses on community invasibility^30^, and the main focus of the present study is to understand how might the magnitudes and timing of resource pulses affect temporal variability of resident communities. For experimental microcosms that received high-magnitude nutrient pulses (i.e., treatment “Early”, “Coincident” and “Late”), the timing of resource pulse events relative to that of introduced model invaders was manipulated. Specifically, the timing of pulsed resources was created either before (i.e., treatment “Early”), coincident with (i.e., treatment “Coincident”), or after the timing of invader arrivals (i.e., treatment “Late”). We did not consider the scenario of low-magnitude supply (i.e., treatment“Multi-pulses”) due to a persistent supply of resource pulses in this treatment group.

The first sampling took place 7 days later after the assembly of resident protist community, and we allowed the experiment to run for 35 days afterward, during which period we sampled each microcosm every 7 days up to the final day of the experiment. On each sampling date, 0.32 ml medium was withdrawn from each well-mixed microcosm for visual counts, and all protist species were identified by their morphological characters. Additionally, 10% of the medium in each microcosm was replaced each week with fresh standard medium to support bacterial growth and reduce the accumulation of metabolic wastes. Because such weekly medium replacement might cause possible background noises, we did the replacement on a daily basis instead (1.4% of the standard medium per day).

Algal and protozoan species were examined and enumerated microscopically based on their morphological characters. We recorded the density of each protist species as the number of individuals per milliliter. Community temporal variability was quantified as the ratio of the standard deviation to the mean of total protist density for each microcosm, with algal and protozoan components separated, which is the reciprocal of community temporal stability^2^. Therefore, the smaller the temporal variability is, the greater the temporal stability is. Also, the degree of population variability for individual species was calculated in a similar way. Community-wide species synchrony (φ_*C*_) was quantified as 
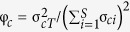
, for which 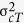 is the variance in total density, and σ_*Ci*_ is the standard deviation in the density of species i in a community with S species^15^,^16^. Accordingly, species asynchrony was calculated as 1 − φ_*C*_, and the measure of species asynchrony is in the range of 0 (perfect synchrony) and 1 (perfect asynchrony), with the relationship between species asynchrony and community temporal variability examined. For dominant resident algal or protozoan species, the measure of dominance was calculated as the relative abundance of representative dominant species in a community, and the correlation coefficients between community temporal stability and dominance were then quantified.

Analysis of variance (ANOVA) procedures were applied to test how temporal variability of resident communities varied under different experimental conditions, and *post-hoc* tests were conducted using the Tukey’s HSD test. Due to variance heterogeneity, a sandwich estimator was included in model analysis to provide heteroscedasticity-consistent estimations of the covariance matrix^42^. All statistical analyses were performed using R (ver. 3.1.3, R Development Core Team 2015).

## Results

### Temporal variability of resident protist community

**Figure 1:**
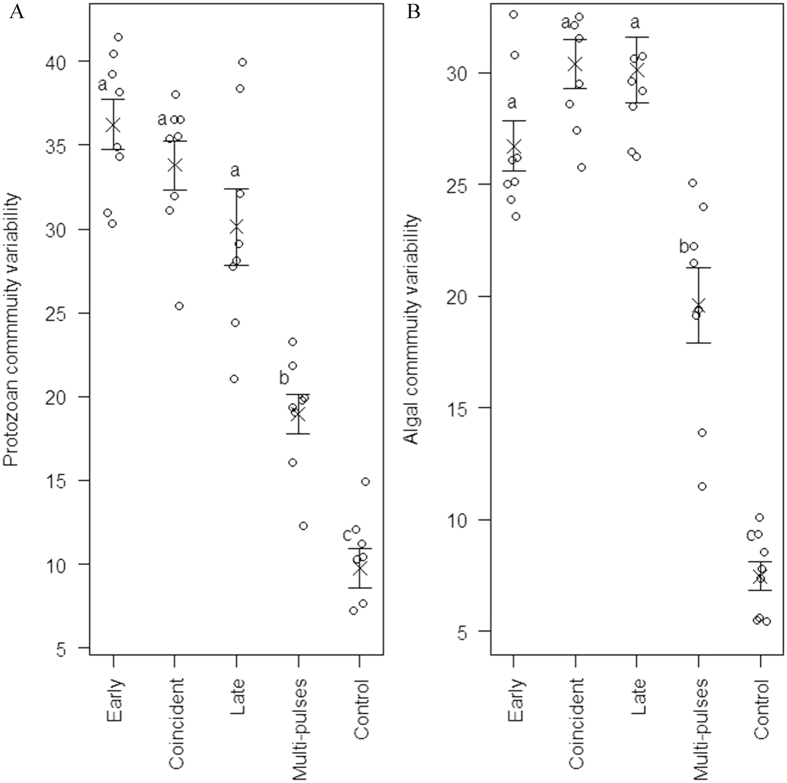
Effects of fluctuating resources on temporal stability of resident (**A**) protozoan and (**B**) algal communities. “Early”, “Coincident” and “Late” are treatments with a concentrated medium supplied at a low frequency (high-magnitude nutrients per pulse), and the timing of nutrient supply varies with the timing that model invaders are introduced to the resident communities. “Multi-pulses” refers to the treatment with the concentrated medium supplied at a high frequency (low-magnitude nutrients per pulse), and “Control” refers to the treatment without adding the concentrated medium. Different small letters indicate statistically significant differences by Tukey’s test. Values are mean ± 1 SE.

**Figure 2:**
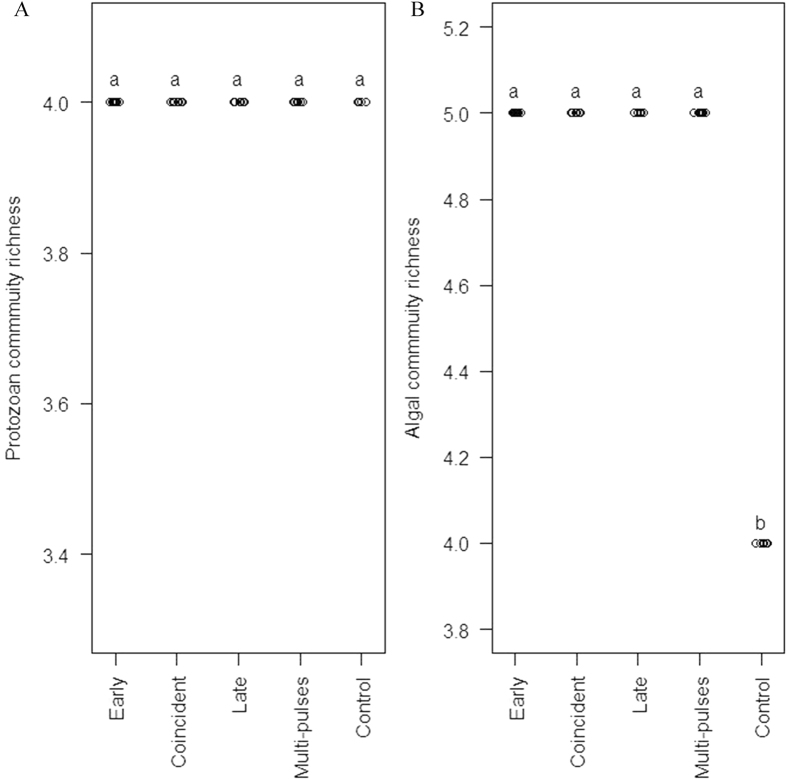
Effects of fluctuating resources on the richness of resident (**A**) protozoan and (**B**) algal communities. Different small letters indicate statistically significant differences by Tukey’s test.

For both protozoan and algal taxonomy component, community temporal variability increased with enhanced resource intensity (protozoan component: F_4,35_ = 49.59, *P* < 0.001; algal component: F_4,35_ = 58.65, *P* < 0.001). Specifically, communities receiving a concentrated medium (treatment “Early”, “Coincident”, “Late” and “Multi-pulses”) displayed higher levels of temporal variability than that of the control group (treatment “Control”; Fig. 1A and B). Meanwhile, community temporal variability increased with enhanced resource pulse magnitude, as communities receiving high-magnitude nutrient pulses (treatment “Early”, “Coincident” and “Late”) were relatively more unstable than communities receiving low-magnitude nutrient pulses (treatment “Multi-pulses”), although the same total amount of the concentrated medium was added to microcosms subjected to any of these four treatments. In contrast, the timing of resource pulses did not significantly affect temporal variability patterns among communities receiving high-magnitude nutrient pulses (treatment “Early”, “Coincident” and “Late”; Fig. 1A and B). Additionally, protozoan richness of resident communities was not significantly altered by fluctuating resource availability (F_4,35_ = 0.74, *P* = 0.58; Fig. 2A), and algal richness showed similar trend except that the control group (treatment “Control”) supported a lower level of richness than that of other groups (F_4,35_ = 209.5, *P *<* *0.001; Fig. 2B).

### Stabilizing mechanisms

**Figure 3:**
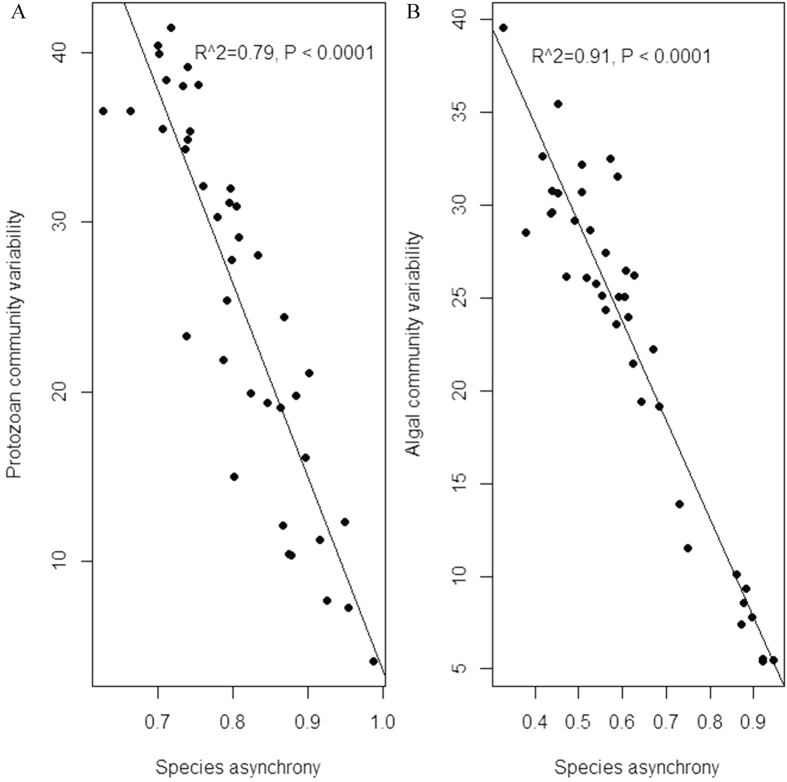
The relationship between community-wide species asynchrony and temporal variability of (**A**) protozoan and (**B**) algal communities. The temporal variability patterns are mainly caused by fluctuating resources.

**Figure 4:**
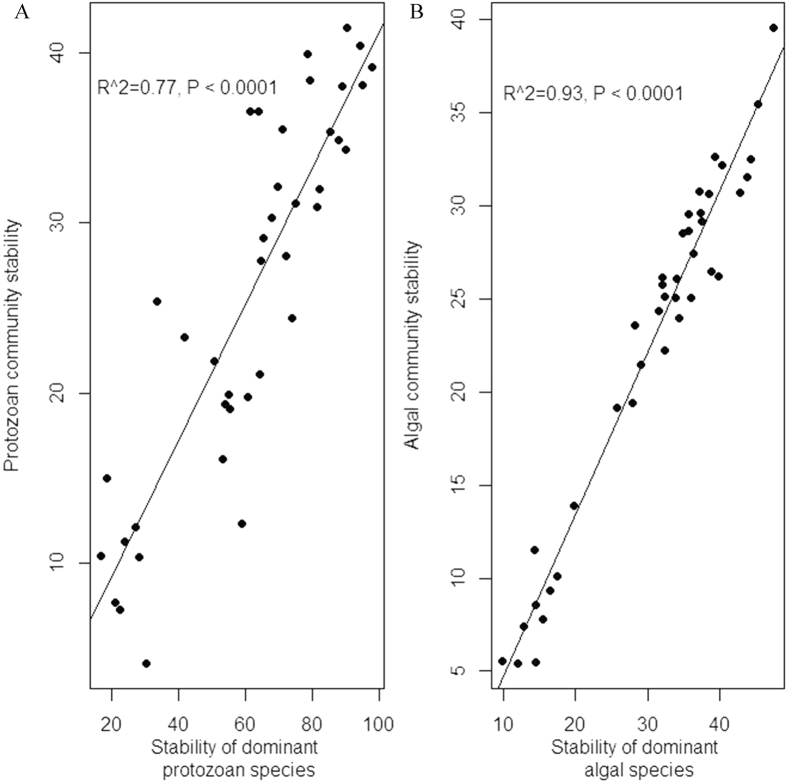
(**A**) The relationship between temporal stability of dominant protozoan species, *C. Striatum*, and temporal stability of protozoan communities; (**B**) The relationship between temporal stability of dominant algal species, *S. opoliensis*, and temporal stability of algal communities.

**Figure 5:**
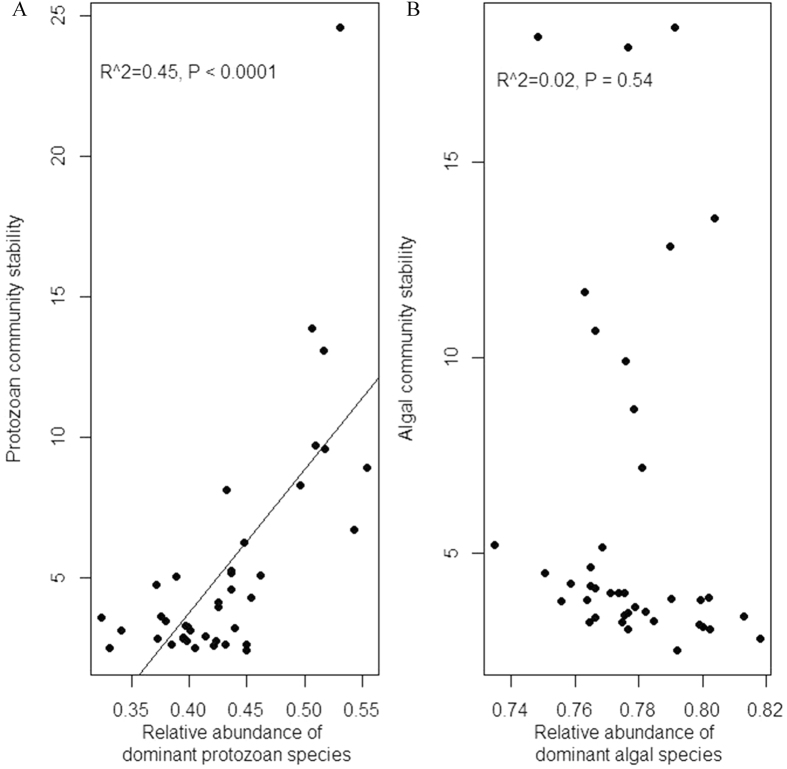
(**A**) The relationship between relative abundance of dominant protozoan species, *C. Striatum*, and temporal stability of protozoan communities; (**B**) The relationship between relative abundance of dominant algal species, *S. opoliensis*, and temporal stability of algal communities.

For both protozoan and algal taxonomy components, community-wide species asynchrony decreased with increasing community temporal variability caused by resource enrichment (Fig. 3A and B). In other words, the degree of species asynchrony and community stability was positively related. The relationship between the stability of protozoan community and that of the dominant protozoan species (i.e., *C. Striatum*), or between the stability of algal community and that of dominant algal species (i.e., *S. opoliensis*), was also positively related (Fig. 4A and B). When the relationship between community stability and dominance was checked, a positive relationship was found for protozoan component (Fig. 5A), but the relationship was not statistically significant for algal component (Fig. 5B).

## Discussion

Consistent with theoretical predictions^5^,^7^ and empirical studies^8^,^10^,^11^,^12^,^43^ that also report a destabilizing effect of nutrient enrichment, our results showed that resident algal and protozoan communities receiving enhanced resource intensity (treatment “Early”, “Coincident”, “Late” and “Multi-pulses”) generally displayed higher levels of temporal variability when compared to the control group (treatment “Control”). Also, communities receiving low-magnitude resource pulses (treatment “Multi-pulses”) displayed lower levels of temporal variability than communities receiving high-magnitude resource pulses (treatment “Early”, “Coincident” and “Late”), which was in congruence with theoretical predictions that small-magnitude resource pulses will stabilize food webs, whereas large-magnitude resource pulse will promote community temporal variability^44^. However, the timing of resource pulses did not significantly affect patterns of community temporal variability among treatment “Early”, “Coincident” and “Late”. One possible explanation is that the effects of species interactions, such as competitive intensity imposed by invaders on resident species, were generally weaker than, and thus masked by the effects of fluctuating resources. Another reason probably lies in the fact that ecological dynamics of the resident community could be affected by initial diversity of protist community^41^, which in our case was similar across treatments. However, the relative importance of resource pulses *versus* species interactions in affecting invasion success might change over time, and future work should address this important question.

Although mounting evidence has shown that high-diversity communities are more stable^2^,^10^,^13^,^14^,^16^,^45^, in the present study resident richness appeared not to be the major factor determining resident community stability, as resident protist richness did not generally vary with resource enrichment. Many studies also reported similar findings that community stability was more affected by factors such as environmental conditions or propagule availability, rather than species richness^12^,^46^,^47^,^48^,^49^. For instance, one empirical study showed that nitrogen-fertilized communities maintained stability despite losses in richness^48^, and another reported that the relationship between diversity and community stability was insignificant after controlling for the effects of N addition^12^. Overall, these studies suggest that environmental context might weaken, or even mask the effect of richness on community stability. In contrast, a simple causal relationship between species richness and community stability is commonly found in studies based on artificially assembled communities. Since multiple interrelated factors are commonly found within more complex natural ecosystems with non-random community assembly, studies that use assembly of artificial communities often tend to overestimate richness effect, but fail to reveal the roles of environmental context and some other key factors that might function as ultimate drivers in determining species richness, community temporal stability and their relationship. Similar problems also exist in studies that address the relationships between species richness and community invasibility. For example, the contradicting patterns between the two (i.e., positive versus negative relationships) might also be reconciled if the roles of the environmental context are explicitly considered^50^,^51^,^52^,^53^.

Since resident protist richness did not play an essential role in influencing community temporal stability, we further tested other possible stabilizing mechanisms. Community-wide species asynchrony is one possible candidate mechanism as asynchronous fluctuations in population dynamics among species tend to offset each other. In line with several other studies^10^,^12^,^14^,^16^,^17^, our results showed a significantly negative relationship between community temporal variability and species asynchrony. Therefore, for experimental microcosms receiving fluctuating resources, algal and protozoan species generally responded to such pulse events in a more similar or synchronous manner. Consequently, such synchronized population dynamics were related to high levels of temporal variability (or low levels of temporal stability), and fluctuating resources might have decreased community stability through its negative impact on the asynchronous responses among species. We also tested another possible mechanism that community stability might be maintained by a single population, especially by the dominant species. Our results showed a positive relationship between the stability of dominant species and the entire protist community, and dominant species should have played an important role in influencing community stability. In particular, the observation that resident communities receiving low-magnitude pulses were more stable than resident communities receiving high-magnitude pulses might be explained by the fact that when the pulse events continued for the entire experiment (i.e., with longer duration), there was more time for the acclimation of the dominant species to the new conditions. As a result, well-adapted dominant species with relatively stable populations could contribute significantly to community stability^21^,^22^. We further tested the relationship between the relative abundance of dominant species and community stability. The results showed that temporal stability of protozoan community increased significantly with the relative abundance of dominant protozoan species, *C. Striatum*, further supporting the importance of dominant species in stabilizing communities (also see refs 12 and 19, 20, 21, 22). However, the lack of a statistically significant positive relationship between community temporal stability and dominant algal species (*S. opoliensis*) suggested that community-wide species asynchrony was more likely to play a vital role in regulating temporal stability of resident algal community.

One caveat in this study is that the created nutrient pulses under laboratory conditions might differ substantially from resource pulses occurring in natural ecosystems in terms of pulse types, magnitudes and duration. Also, the selection of model invaders was arbitrary as physiological and ecological properties of selected organisms might affect experimental results. Additionally, this study was mainly bottom-up oriented, and did not consider the role of consumers in regulating community dynamics, which might be important as consumers may stabilize ecological communities through enhancing weak trophic interactions^54^. However, our study results may still be of general relevance in situations where ecological communities are exposed to natural resource pulses. Actually, the phenomena of cross-ecosystem resource subsidies are commonly observed^55^,^56^, and several empirical studies report that such pulse events exert strong influence over community dynamics. For example, increased inputs of terrestrial carbon could decrease the stability of recipient aquatic ecosystems^57^. Similarly, island-inhabiting producers and consumers receiving nutrient pulses from seabird guano were more variable than their counterparts from islands that were not subsidized^58^. Also, the deposition and decomposition of insect (e.g., cicada) or fish carcasses (e.g., salmon, carp) could alter the temporal variability of studied systems, although the consequences of such pulses are contingent on a multitude of factors, such as the quality, magnitudes, timing and duration of resource pulses, organism trophic levels, as well as the characteristics and sensitivity of recipient communities^23^,^56^,^59^,^60^. Obviously, to better understand the complex ecological consequences of natural resource pulses, especially the effects of resource subsidies across habitat and ecosystem boundaries on the stability and functioning of recipient ecosystems, more theoretical and empirical studies are needed.

Since resource pulses are widespread phenomena, and many invasive species are commonly found in habitats with fluctuating nutrient supplies, we should closely monitor these habitats, and take effective approaches to prevent the establishment and spread of such species once their footprints are spotted. In particular, for those habitats experiencing large pulses of anthropogenic nutrient inputs, mandatory nutrient reductions are needed to reduce the windows of opportunities for invasive species. Meanwhile, ecologists have sought to develop a robust, general theoretical framework that integrates a large number of hypotheses of invasion ecology, and thus provide a synthetic approach to advancing its development^61^,^62^,^63^,^64^. Resource-based approach could help develop such a comparative framework that incorporates aspects of population dynamics, species interactions and community properties (e.g., community temporal stability and invasibility), given that resource impacts and requirements are key components underlying many ecological processes^26^,^51^,^65^,^66^. More details related to fluctuating resources, such as pulse magnitude, pulse duration and the timing of such events, should be included for the further development of such resource-based approach.

Overall, the current study showed that fluctuating resource availability increased community temporal variability. Specifically, the magnitude and duration, rather than the timing of pulse events, significantly influenced temporal variability patterns of resident communities. Resident protist richness was not the major driver of temporal stability of resident communities. Instead, resource pulses reduced community stability by increasing the degree of community-wide species synchrony and decreasing the stabilizing effects of dominant protist species. Therefore, studies that explicitly test how the changing environmental content could affect community stability and the regulatory mechanisms involved deserve more attention, and this is especially true when diverse ecosystems are facing with increasing anthropogenic perturbations.

## Additional Information

**How to cite this article:** Li, W. and Stevens, M. H. H. Community temporal variability increases with fluctuating resource availability. *Sci. Rep.* **7**, 45280; doi: 10.1038/srep45280 (2017).

**Publisher's note:** Springer Nature remains neutral with regard to jurisdictional claims in published maps and institutional affiliations.
